# γ-AApeptides–based Small Molecule Ligands That Disaggregate Human Islet Amyloid Polypeptide

**DOI:** 10.1038/s41598-019-56500-0

**Published:** 2020-01-09

**Authors:** Olapeju Bolarinwa, Chunpu Li, Nawal Khadka, Qi Li, Yan Wang, Jianjun Pan, Jianfeng Cai

**Affiliations:** 1 Department of Chemistry, University of South Florida, 4202 East Fowler Avenue, Tampa, Florida 33620 United States; 2 Department of Medical Oncology, Shuguang Hospital, Shanghai University of Traditional Chinese Medicine, Shanghai, 201203 P. R. China; 3 Department of Physics, University of South Florida, 4202 East Fowler Avenue, Tampa, Florida 33620 United States

**Keywords:** Biochemistry, Diseases, Chemistry, Chemical biology, Peptides

## Abstract

The abnormal folding and aggregation of functional proteins into amyloid is a typical feature of many age-related diseases, including Type II diabetes. Growing evidence has revealed that the prevention of aggregate formation in culprit proteins could retard the progression of amyloid diseases. Human Amylin, also known as human islet amyloid polypeptide (hIAPP), is the major factor for categorizing Type II diabetes as an amyloid disease. Specifically, hIAPP has a great aggregation potential, which always results in a lethal situation for the pancreas. Many peptide inhibitors have been constructed from the various segments of the full-length hIAPP peptide; however, only a few have their origin from the screening of combinatorial peptidomimetic library. In this study, based on HW-155, which was previously discovered from a one–bead–one compound (OBOC) library to inhibit Aβ_40_ aggregation, we investigated eight (8) analogues and evaluated their amyloid-prevention capabilities for inhibiting fibrillization of hIAPP. Characterization studies revealed that all analogues of HW-155, as well as HW-155, were effective inhibitors of the fibril formation by hIAPP_._

## Introduction

The human islet amyloid polypeptide (hIAPP), also known as amylin, is a unique amyloidogenic precursor peptide, and a critical pathogenic biomarker of Type II diabetes. Amylin is co-secreted with insulin by the β-cells of the pancreas, and co-stored in the secretory granules^1^. There is overwhelming evidence of the useful role of soluble amylin in glucose metabolism. However, the insoluble aggregates resulting from the misfolding of this protein are implicated in type II diabetes^2–4^. Just like the amyloid deposits formed in neurodegenerative disorders (e.g., Alzheimer’s, Parkinson’s and Huntington’s diseases)^5^ and progressive diseases (e.g., type II diabetes and cystic fibrosis)^6,7^, amylin aggregates consist of stacked protofilaments, leading to a structural architecture known as the cross-β structure^8–10^. The oligomerization of amylin into amyloid fibrils has been suggested to be a stepwise process: first, the aggregation of monomers into colloidal spheres (nucleation units), which stop growing after reaching a threshold diameter; second, the association of the spheres to form linear chains of toxic, mature fibrils of pore-like morphology^8^. The extent of this oligomerization process is strongly correlated to pancreatic β-cell dysfunction and death in Type II diabetic patients^11–13^; therefore, an impairment of the initial monomer aggregation process is an appealing therapeutic approach to diabetes drug discovery and development.

To date, there is no cure for pancreatic amyloidosis, and there are only limited approved therapeutic strategies for its prevention^14^. Hence, there is an urgent need for potent inhibitors of the process producing these pathogenic fibrils. In line with this, some inhibitors, including the peptides derived from the various secondary recognition elements along the amylin peptide chain^15–17^, engineered peptides^18–20^, constrained peptides^21–27^, small molecules^28,29^ and natural products^30–32^, have been reported. Many of these inhibitors are specific in their activity; however, a good number of them have exhibited dual inhibitory activity such that they can inhibit amyloid aggregation of multiple classes of proteins non-specifically^32–35^.

**Figure 1:**
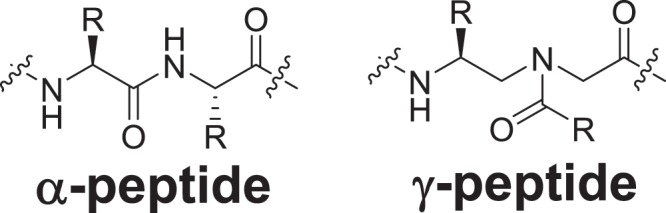
A dipeptide motif of α-peptide and a monomeric unit of γ-AApeptide.

In order to advance the application of peptidomimetics in chemical biology and drug discovery, we have recently introduced a new class of peptidomimetic scaffold called γ-AApeptides (Fig. 1)^36,37^. γ-AApeptides contain *N*-acylated-*N*-aminoethyl amino acid units, and are derived from γ-PNAs^38^. Each monomeric unit of γ-AApeptides is equivalent to a dipeptide motif in a conventional α-peptide; thus a γ-AApeptide can project an identical number of functional groups as an α-peptide of the equal length. γ-AApeptides have been shown to form well-defined helical structures^39–41^, target bacterial membranes^42–44^, and specifically bind to protein surfaces to modulate protein functions and cellular activities^45–48^.

**Figure 2:**
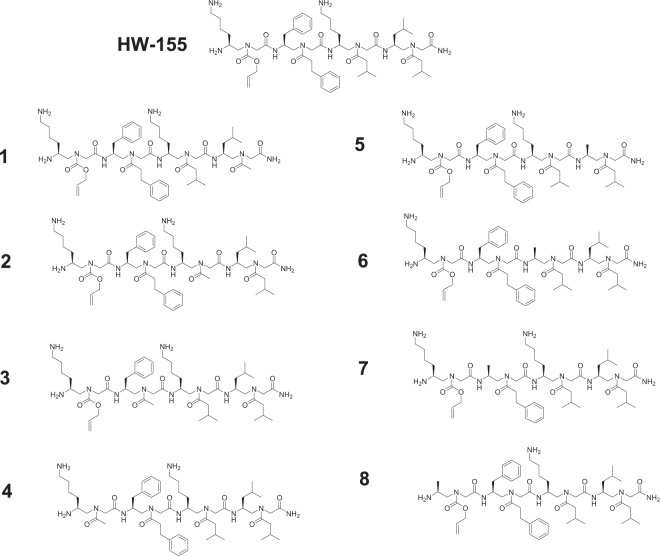
Peptide sequences of **HW-155** and its analogues **1–8**.

In our previous work, we reported a small molecular inhibitor of Aβ_40_ aggregation, HW-155, which was identified from an OBOC (one-bead-one-compound) γ-AApeptide combinatorial library (Fig. 2)^45^. **HW-155** is a γ-AApeptide^38^ tetramer (comparable to an 8-mer peptide in length) with little structural similarity to KLVFF, the core amyloid-forming unit of Aβ. Inspired by the findings on molecules that show cross-inhibition toward multiple amyloidosis^32–35^, and based on the fact that **HW-155** was identified from an OBOC library, we hypothesized that **HW-155** could be a non-specific amyloid inhibitor, and as such it may exhibit inhibitory activity toward the aggregation of hIAPP. To test our hypothesis, we synthesized **HW-155** and eight (8) analogues (**1–8**) with methyl group substitution on each position (similar to an alanine scan), and investigated their ability to prevent the fibrillization of amylin (hIAPP), as well as to disrupt preformed hIAPP aggregates. Furthermore, we studied the aggregation behaviour and morphological characteristics of hIAPP in the presence and absence of various concentrations of HW-155 and its eight (8) analogues using transmission electron microscopy (TEM), time-dependent Thioflavin T (ThT) fluorescence assay, and atomic force microscopy (AFM). To further explore the biological potential of **HW-155** and its analogues (Fig. 2), we examined their inherent toxicity and the ability to improve the viability of hIAPP-treated cells.

## Results and Discussion

### Prevention of hIAPP aggregation

**Figure 3:**
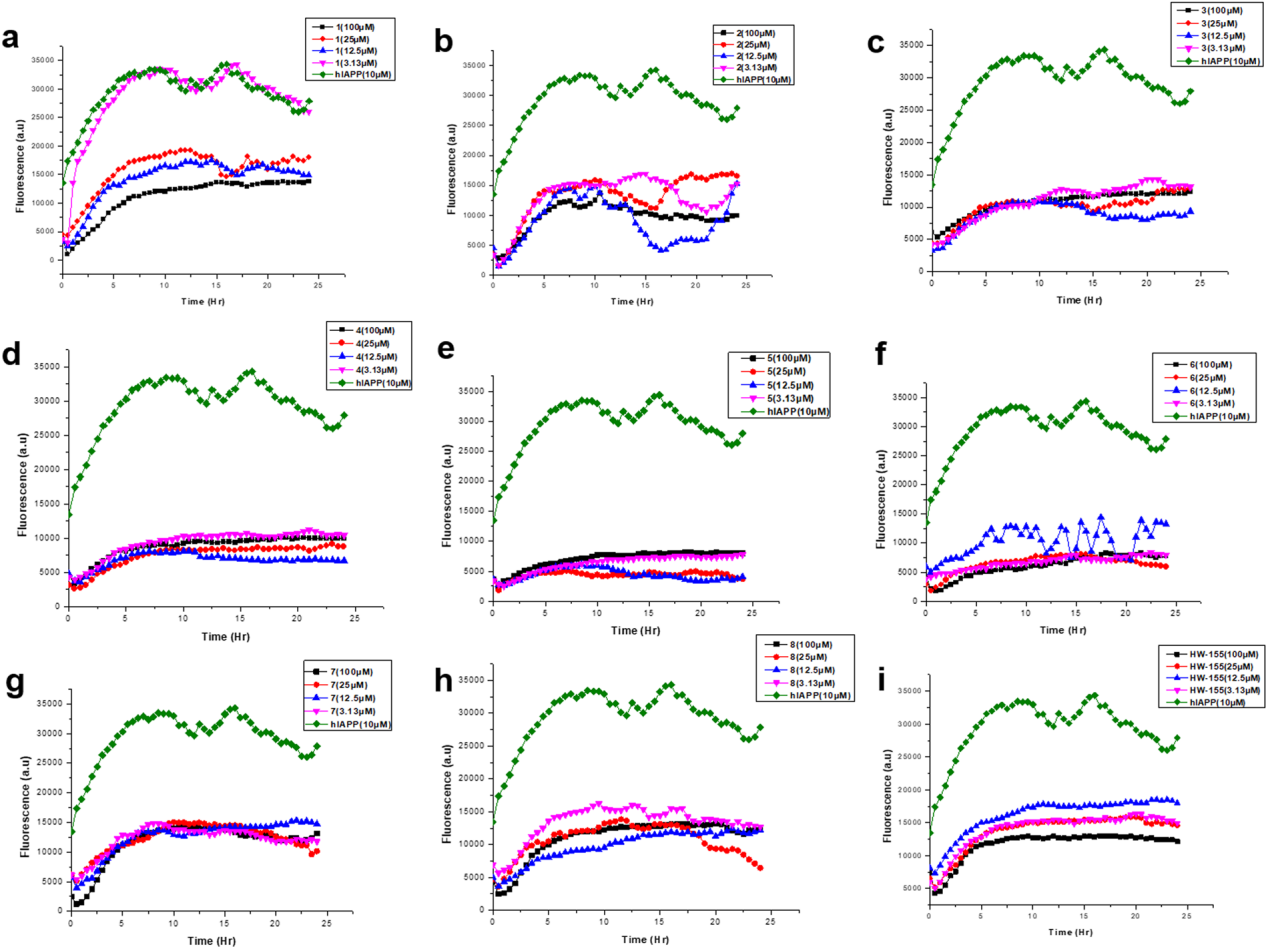
Time-dependent ThT assay of hIAPP (10 µM) and various molar equivalents of **HW-155**, and **1–8**.

We have previously reported a γ-AApeptide oligomer, **HW-155**, as a potent inhibitor of Aβ aggregation. In this study, we first investigated the effectiveness of **HW-155** in the inhibition of amyloid formation by hIAPP. Although ThT assay is rather qualitative than quantitative, it was first carried out to preliminarily assess the inhibitory effect of HW-155. Time-dependent ThT assay revealed a significantly enhanced fluorescent intensity with hIAPP (10 µM) alone (Fig. 3). Surprisingly, at a concentration as low as 3.13 µM, **HW-155** inhibited hIAPP aggregation by about 50%, whereas 10- and 2.5-fold molar excesses of **HW-155** were able to reduce hIAPP aggregation to 42% and 50%, respectively (Fig. 3, Table 1). These observations suggest that **HW-155** could not only inhibit Aβ aggregation, but also prevent the amyloid formation of hIAPP.

**Table 1:** Percentage of 10 µM hIAPP aggregation (based on ThT Fluorescence assay) in the presence of **HW-155** and analogues **1–8** at different concentrations.

Peptides	(%) aggregation			
	100 µM	25 µM	12.5 µM	3.13 µM
1	43.5	57.5	53.5	45.2
2	34.0	48.7	28.4	47.0
3	38.8	36.1	30.5	42.4
4	32.0	27.9	23.1	34.6
5	26.1	14.7	13.9	24.0
6	24.0	23.8	36.0	24.6
7	43.4	44.2	46.7	42.4
8	41.9	37.2	37.8	47.3
HW-155	42.0	50.4	59.0	51.2

Inspired by the preliminary data, we sought to understand the relationship between the structure of **HW-155** and its inhibitory activity against hIAPP aggregation (i.e., structure and function relationship). Using **HW-155** as a lead sequence, we designed and performed a functional analysis of eight (8) analogues of **HW-155**, namely γ-AApeptides **1–8** (Fig. 2). For four analogues **(1–4)**, we substituted each of the *N*-side chains in **HW-155** with an acetyl group, while alanine scanning was performed on the chiral side chains **(5–8)**.

Amyloid aggregation kinetics of hIAPP (10 µM) in the presence of **1–8** was monitored by time-dependent ThT assay. In an initial attempt, we studied the anti-aggregation activity of 2.5-, and 10-fold molar excesses of **1–8** against hIAPP aggregation. Interestingly, peptides **2–8** were able to suppress the ThT fluorescence induced by hIAPP aggregation by about 50% or more, while, peptide **1** suppressed ThT fluorescence induced by hIAPP aggregation by at least 43% (Table 1). Further investigation of nearly equimolar or lesser concentrations of **1–8** revealed a minimum aggregation inhibition of at least 50% even at the lowest concentration of 3.13 µM (Fig. 3).

Intriguingly, we identified that peptides **3**, **4**, **5**, and **6** (Table 1, Fig. 3c–f) could inhibit more than 55% of hIAPP aggregation (with less than 45% aggregation found) across all molar equivalents tested. In fact, **5** (Fig. 3e) reduced the extent of hIAPP aggregation to as low as 14% even at a nearly equimolar concentration (12.5 µM). The inhibition assay indeed showed a good structure function relationship. For instance, it seems that achiral chains (introduced through acylation) near C-terminal end are not critical, as both peptide **1** and **2** resulted in similar anti-aggregation activity as the parent compound, **HW-155**. However, change to acetyl groups (**3** and **4**) improved the aggregation inhibitory activity. In the achiral side chain truncation series, peptide **4** emerged with the best anti-aggregation activity with almost 2-fold decrease in hIAPP aggregation across the concentrations tested compared to **HW-155**.

Interestingly, conversion of the chiral side chain (derived from α-side chain) in **HW-155** into methyl groups (**5–8**) gave rise to a better or similar inhibitory activity than **HW-155**. However, unlike acetylation, which seems to be more critical in inhibition near the N-terminal end, mutation to methyl side chain played a more important role near the C-terminal end, as evidenced by **5** and **6**. As a matter of fact, for all the synthesized analogues, **5** exhibited the best anti-aggregation potential with almost 3–4 fold inhibition against hIAPP aggregation.

Given the promising inhibition study by ThT assay, we next carried out TEM experiment to further confirm the ability of the few most potent compounds to inhibit hIAPP aggregation. This is done by pre-incubating nearly equimolar amount of peptides **2, 3, 4, 5** and **HW-155** with hIAPP.

**Figure 4:**
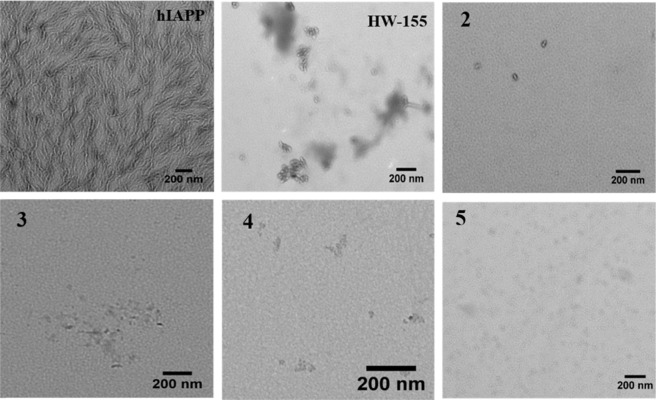
TEM images of hIAPP alone and in the presence of nearly equimolar amount **2, 3, 4, 5**, and **HW-155**.

The TEM micrographs for hIAPP revealed a dense network of intertwined mature fibrils with linear, and thread-like morphology (Fig. 4), indicating the presence of hIAPP amyloid. The presence of fibrillar morphology under the electron microscope is a characteristic feature of amyloidogenic peptides^49^. However, no such fibril was observed in the presence of **2**, **3**, **4**, **5**, and **HW-155**, indicating a significant prevention of fibril formation by hIAPP. A prevention of fibril formation by **2** and **5** resulted in monomers as observed in the TEM images. Likewise, oligomers seen in **3**, **4**, and **HW-155** were a result of the inhibition of mature fibril formation.

### Disaggregation of hIAPP fibrils

**Figure 5:**
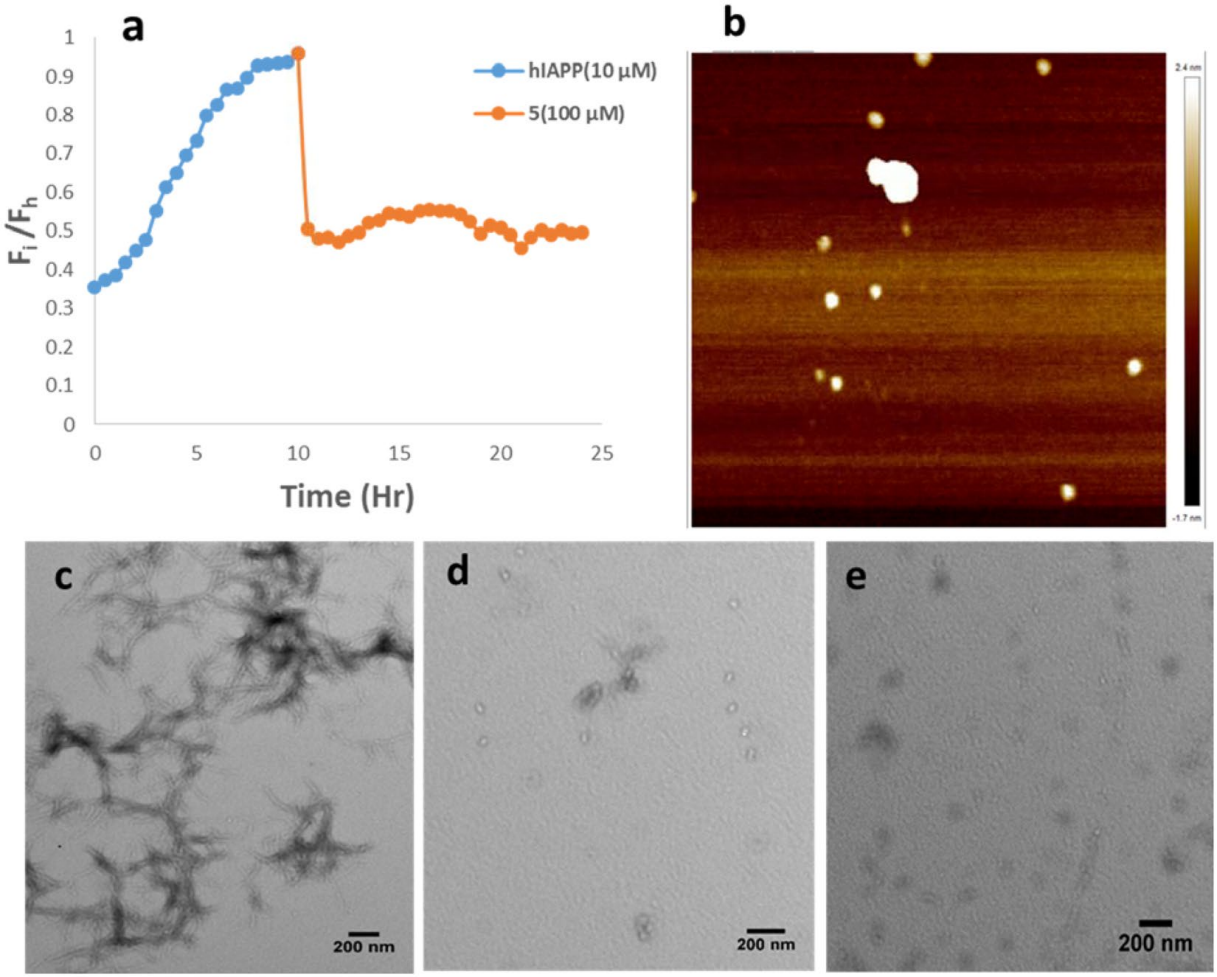
(**a**) Time-dependent ThT assay of the disruption of hIAPP (10 µM) preformed fibrils by **5** (100 µM). (**b**) AFM image the disruption of hIAPP (10 µM) preformed fibrils by 5 (100 µM). (**c–e**) TEM images of aged hIAPP fibrils treated with buffer (**c**) and 100 µM peptide **5** (**d,e**).

To evaluate the ability of our designed analogues to disaggregate preformed hIAPP fibrils, we performed a time–dependent ThT assay and monitored the fluorescent intensity for up to twenty-four (24) h. Interestingly, only **5** was able to disrupt preformed hIAPP fibrils at a tested concentration of 100 µM (Fig. 5a).

To visualize the effect of **5** on hIAPP preformed aggregates, we allowed 10 µM hIAPP to age for 10 h before treatment with a 10–fold molar excess of **5**. AFM showed a disappearance of mature fibrils (Fig. 5b). However, very few oligomeric hIAPP spheres resulting from the disrupted aggregates were observed. This confirms that **5** is able to disaggregate preformed hIAPP aggregates. TEM images also corroborate this finding as only monomeric and oligomeric units were observed with peptide **5-**treated hIAPP (Fig. 5d,e). On the contrary, hIAPP that was treated with equal volume of buffer (control) revealed a network of fibrillar aggregates (Fig. 5c).

### Cell toxicity studies

**Figure 6:**
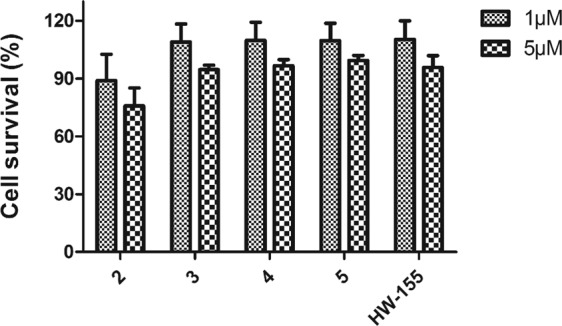
Effects of peptides **2**, **3**, **4**, **5**, and **HW-155** on NIH-3T3 cells in CCK-8 cell viability assay.

Based on our findings of the inhibition of hIAPP fibrillization by **2, 3**, **4**, **5**, and **HW-155**, we further assessed their safety index in mammalian cells by CCK-8 assay. We tested the toxic effects of **2, 3**, **4**, **5**, and **HW-155** on NIH-3T3 cells (Fig. 6). At 1 µM concentration, only peptide **2** significantly altered the cell viability (~10% reduction). However, at a peptide concentration of 5 µM, peptides **3, 4, 5**, and **HW-155** reduced the cell viability by at least 10%, while peptide **2** showed a significant cell viability reduction of about 20%. At both concentrations tested, peptide **2** exhibited a mild cytotoxic effect on NIH-3T3 cells.

**Figure 7:**
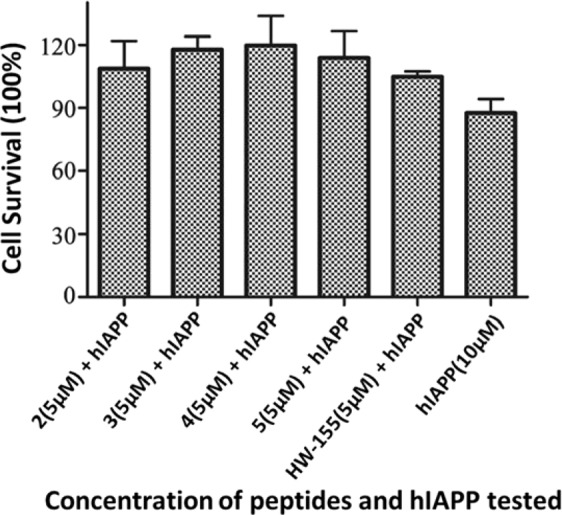
Evaluation of the potential of peptides **2, 3, 4, 5**, and **HW-155** to improve the cell survival in the presence of hIAPP amyloid.

To study the ability of peptides **2, 3, 4, 5**, and **HW-155** to modulate the hIAPP amyloid–induced cytotoxicity, we treated NIH-3T3 cells with hIAPP (10 µM) and 5 µM peptide as described in the general information section. Results showed that cells treated with only hIAPP exhibited a significant reduction in survival, whereas treatment with 5 µM peptides **2, 3, 4, 5**, and **HW-155** was able to rescue the cells from the hIAPP amyloid cytotoxicity (Fig. 7). It is noteworthy that peptides **3, 4**, and **5** were better at reducing cell death due to hIAPP amyloid than **2** and **HW-155**.

## Discussion

We have previously reported an inhibitor of Aβ aggregation by HW-155 from combinatorial screening. The possibility of cross inhibitory activity of amyloid inhibitors^32–35^ toward multiple classes of protein fibrils prompted our effort to investigate its inhibition of hIAPP aggregation. To probe the importance of side chains on the aggregation of hIAPP, we synthesized a few derivatives of **HW-155**. Consistent to our hypothesis, **HW-155** is a non-selective inhibitor of amyloid proteins as it also inhibited the aggregation of hIAPP. Interestingly, replacement of side chains of **HW-155** to methyl groups lead to sequences which either retained or better inhibited hIAPP fibril formation. It may suggest that none of the positions on the molecular scaffold is crucial for the inhibition of hIAPP aggregation. All the groups may function together to prevent the growth of the amyloid fibrillation. We also noted that only peptide **5** was able to disassemble preformed hIAPP fibrils. The mechanism is not clear at this point. We postulate it may be because after amyloid fibril formation, the hIAPP molecules are packed tightly. In order to disrupt the formed aggregation, the inhibitor has to insert into the space between adjacent molecules in the aggregates, therefore the orientation of the groups on the inhibitor has to be restricted to facilitate the molecular insertion. In addition, hIAPP fibril formation is known to induce certain cytotoxicity to cells; it is encouraging to observe that **HW-155** and other derivatives could alleviate the cytotoxic effect of hIAPP and rescue cell survival at certain level of extent. We believe this is a strong supporting proof that these peptidomimetics prevented the fibrillar formation of hIAPP in the cell culture.

## Conclusion

Our previous and present studies provided experimental support to the dual action of **HW-155** in preventing amyloid fibril formation by Aβ^45^ and hIAPP_._ We have utilized a simple, but effective approach for designing potential inhibitors of hIAPP fibrillization. We have also demonstrated the ability of **HW-155** analogue, peptide **5**, to effectively disaggregate preformed hIAPP fibrils. The truncation/substitution of side chains in **HW-155** resulted in analogues that were shown to be more effective in preventing amyloid formation and disaggregating preformed hIAPP amyloids than **HW-155. HW-155** is a dual inhibitor compound; it has a greater advantage over other known amyloid inhibitors that only target a specific amyloid species. Our study also show that **HW-155** and its analogues could provide a useful template for designing potent analogues that could be next-generation therapeutic agents for the treatment of Type II diabetes mellitus.

## Methods

### Peptides synthesis

#### General information

Fmoc–protected α-amino acids and Rink amide resin used for γ-AApeptide synthesis were purchased from Chem-Impex International, Inc. All chemicals and solvents used were purchased from Aldrich or Fisher and were used without further purification.

### γ-AApeptide monomer synthesis

#### Solid phase peptide synthesis

**Figure 8:**
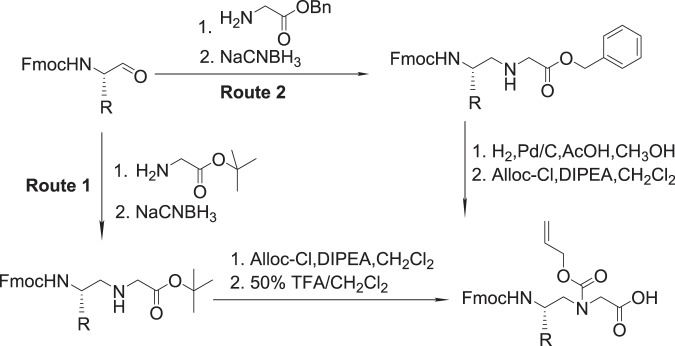
Synthesis of γ-AApeptide building block/monomeric unit.

Peptides were synthesized on the rink amide resin (0.6 mmol/g) on a Burrell Wrist-action shaker. 200 mg resin was treated twice (15 mins each time) with 3-mL 20% Piperidine in DMF to deprotect the Fmoc group. The beads were then washed twice with 3 mL each of DCM and DMF. The desired *N*-alloc protected γ-AApeptide building blocks were prepared (Fig. 8) and are combined (2 equivalents) with 4 equivalents each of HOBt and DIC in 3 mL DMF. The solution was then added to the deprotected resin and was allowed to react for 4 h. Ninhydrin test^38^ was used to confirm the success of the coupling and depending on the Ninhydrin test result, a second round of coupling was done. On reaction completion, the beads were washed with DMF and DCM, followed by a fifteen minutes (15 min) capping reaction with 500 uL Acetic Acid. After capping, the beads were washed and alloc-deprotection reaction was done by reacting the beads with Pd (PPh_3_)_4_ (1 equivalent) and Me_2_NH BH_3_ (6 equivalents) (10 mins each time). The beads were then washed with DCM and DMF, followed by the reaction of the deprotected beads with acyl chloride (4 equivalents) and DIPEA (6 equivalents) in 3 mL DCM for 1 h or with the carboxylic acid (4 equivalents), HOBt (8 equivalents), and DIC (8 equivalents) for 6 h (x2).

**Figure 9:**
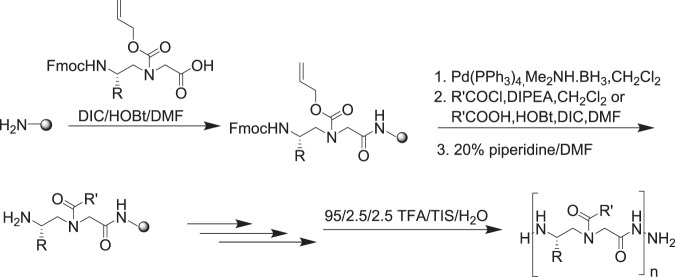
Solid Phase synthesis scheme for **HW-155** and **1–8**.

The above steps were repeated to assemble the desired sequence on the resin (Fig. 9). After these, the resin was washed and the peptide was cleaved from the resin with the cocktail: TFA/H2O/TIS (95/2.5/2.5) for 2.5 h. The solvent was evaporated to obtain the crude peptide, which was analysed and purified on an analytical (1 mL/min) and a preparative (20 mL/min) Waters HPLC system, respectively. 5–100% linear gradient of 0.1% TFA/ACN in 0.1% TFA/H2O over 50 min was used. The HPLC traces were detected at 215 nm. The products were confirmed by MALDI-TOF MS. The product fractions were then collected and lyophilized.

### hIAPP sample preparation

This was done according to previously published protocol^25,50^. 3.1 mg of hIAPP was dissolved in minimal amount of HFIP (50 uL) to remove aggregated hIAPP and the HFIP was removed over a gentle stream of Nitrogen gas. The process was done twice and 5 mL of Milli Q pure water was added to the disaggregated hIAPP film. The solution was then divided into 25 aliquots of 200 µL each. The 25 aliquots were lyophilized and reconstituted in 800 µL of PBS prior to use to obtain a hIAPP stock solution of 40 µM (see Table 2 below). The stock solution was always sonicated prior to use.

**Table 2:** Calculation of hIAPP concentration.

Steps	Calculations
Converting 3.1 mg hIAPP to moles	$\frac{0.0031\,{\rm{g}}}{3904.5\,{\rm{g}}\,/\,{\rm{mol}}}=0.79\,\mu {\rm{mole}}$
Concentration of hIAPP dissolved in Milli Q pure water	$0.79\,\mu {\rm{mol}}/0.005\,{\rm{L}}=158.8\,\mu {\rm{M}}$
Each of the 25 aliquots of 200 µL contains	158.8 µM × 200 µL = 0.0318 µmol of lyophilized hIAPP
Reconstitution of lyophilized hIAPP	$0.0318\,\mu mol\,hIAPP/800\,\mu L=39.75\,\mu M= \sim \,40\,\mu {\rm{M}}$

### Thioflavin T fluorescence assay

#### Inhibition of hIAPP aggregate formation

Different peptide concentrations were made in PBS (1x, pH 7.4). hIAPP stock solution (40 µM) was also prepared as described above. The peptide, hIAPP and 15 µM ThT were added into a 96-well black plate. Equal volume of hIAPP solution was added into the 96-well black plate to make the final concentration of hIAPP in each well to be 10 µM. Time-dependent fluorescent change was monitored for 24 h by a Biotek Synergy H1 hybrid plate reader at excitation and emission wavelengths of 435 and 490 nm, respectively.

#### Disruption of preformed aggregates

Similar to the protocol described above for the inhibition studies, hIAPP and ThT were added to PBS in a 96-well black plate and at concentrations of 10 and 15 µM, respectively. Time dependent fluorescent change was monitored for 10 h, after which 5 uL peptide was added to make a final peptide concentration of 100 µM. 5 µL PBS was also added for control wells. Each test well containing the peptide has a control well to which equal volume of PBS was added. The fluorescent change was then monitored for another 12 h. The relative Fluorescence, Fi/Fh was then plotted against the time (Hr),where *Fi* is the fluorescent intensity of the hIAPP treated with **5** at time t, *Fh* is the Fluorescent intensity of the hIAPP treated with PBS(pH 7.4,1X) at time t.

### Transmission electron microscopy (TEM)

Sample preparation was similar to ThT assay. Sample solution containing 10 µM hIAPP and 12.5 µM of the respective peptide were incubated at 30 °C for 24 h. 10 µL aliquot of the incubated solution was placed on a carbon-coated 200-mesh copper grid. After 10 mins, excess solution was wicked away and the grid was allowed to dry. This is then followed by the addition of 2% uranyl acetate solution (10 µL), which was allowed to float for 5 min. The excess solution was then removed using the blotting paper. The copper grid was left to dry at room temperature before imaging with a FEI Morgagni 268D TEM operated at 60 kV.

### Atomic force microscopy (AFM)

A droplet (10 µL) of the sample solution was placed on a freshly cleaved mica disk and dried in open air at room temperature. Imaging was performed using a Multimode 8 AFM (Bruker, Santa Barbara, CA) and a Nanoscope V controller in the Peak-Force quantitative nanomechanics (QNM) mode (in air). A silicon cantilever probe with a spring constant of 2.8 N/m was used for imaging at room temperature (scan rate of ~1 Hz). The acquired images were leveled by subtracting a linear background.

### Percentage aggregation

The percent aggregation was calculated using the formula below:

Percent aggregation = (F in the presence of inhibitor/F in the absence of inhibitor) × 100%, where F is the average of the ThT signals  from 600 min onwards. These time points were chosen because the maximum ThT signals were observed at these points in the uninhibited hIAPP control experiment.

### Cell viability/proliferation assay

Mouse embryonic fibroblasts NIH-3T3 cell were purchased from Lifetechnoglies. The NIH-3T3 cell were grown at 37°C and 5% CO2 humidified atmosphere in DMEM medium, respectively, supplemented with 10% (v/v) heat-inactivated fetal calf serum, 2 mM glutamine, 100 units/ml penicillin, and 100 mg/ml streptomycin (Invitrogen, Carlsbad, CA). Cell proliferation was determined using the CCK-8 cell counting kit (Sigma- 41 Aldrich). Cells were seeded in 96-well plates at 1 × 10^4^ cells/well. When the cells reached 60% confluence, the cell culture medium in each well was then replaced with 100 μL of cell growth medium containing peptides **2, 3, 4, 5**, and **HW-155** alone at concentrations of 1 or 5 µM peptide with 10 µM hIAPP_._

After incubation for 24 h at 37 °C, the peptides were washed with PBS three times. Then, 10 μL of CCK-8 dye and 100 μL of DMEM cell culture medium were added to each well, and the cells were incubated for another 1.5 h at 37 °C. The absorbance at 450 nm was measured by a Synergy H1 Hybrid Reader (BioTek, Dallas, TX, USA). Untreated cells served as controls with 100% viability. The results are presented as the mean ± SD of three measurements.

## Supplementary information

Supplementary materials.
