# Supplementary material for: γ-AApeptides–based Small Molecule Ligands That Disaggregate Human Islet Amyloid Polypeptide
Source: Sci Rep. 2020 Jan 9;10:95. doi: 10.1038/s41598-019-56500-0 (PMC6952368; doi:10.1038/s41598-019-56500-0)
Supplement: Supplementary file 1 — Supplementary materials. [file 41598_2019_56500_MOESM1_ESM.pdf]

γ-AApeptides–based Small Molecule Ligands That Disaggregate Human Islet Amyloid Polypeptide

Olapeju Bolarinwa,[1] Chunpu Li,[1,2] Nawal Khadka,[3] Qi Li,[2] Yan Wang,[2] Jianjun Pan,[3,*] and Jianfeng Cai[1,*]

[1]Department of Chemistry, University of South Florida, 4202 East Fowler Avenue, Tampa, Florida 33620, United States.

[2]Department of Medical Oncology, Shuguang Hospital, Shanghai University of Traditional Chinese Medicine, Shanghai 201203, P. R. China

[3]Department of Physics, University of South Florida, 4202 East Fowler Avenue, Tampa, Florida 33620, United States.

jianfengcai@usf.edu and panj@usf.edu

Supplementary Information

Table S1 HPLC purities and retention time of peptides 1-8 and HW-155

Peptide	Molecular Mass [M+H]+ (expected/observed)	Purity (based on HPLC) %	Retention Time (min)	Yield (%)
**1**	1048.6845/1048.6747	97.73	18.38	80
**2**	1048.6845/1048.6746	98.95	19.64	83
**3**	1000.6845/1000.6750	99.90	17.49	70
**4**	1048.7209/1048.7217	99.98	18.74	78
**5**	1048.6845/1048.7781	95.82	18.01	90
**6**	1033.6736/1033.7318	98.13	21.54	87
**7**	1014.7001/1014.7594	99.98	17.78	95
**8**	1033.6736/1033.7430	99.02	20.58	96
**HW-155**	1090.7314/1090.7928	98.56	18.95	75

Figure S1 HPLC spectra of peptides 1-8 and HW-155

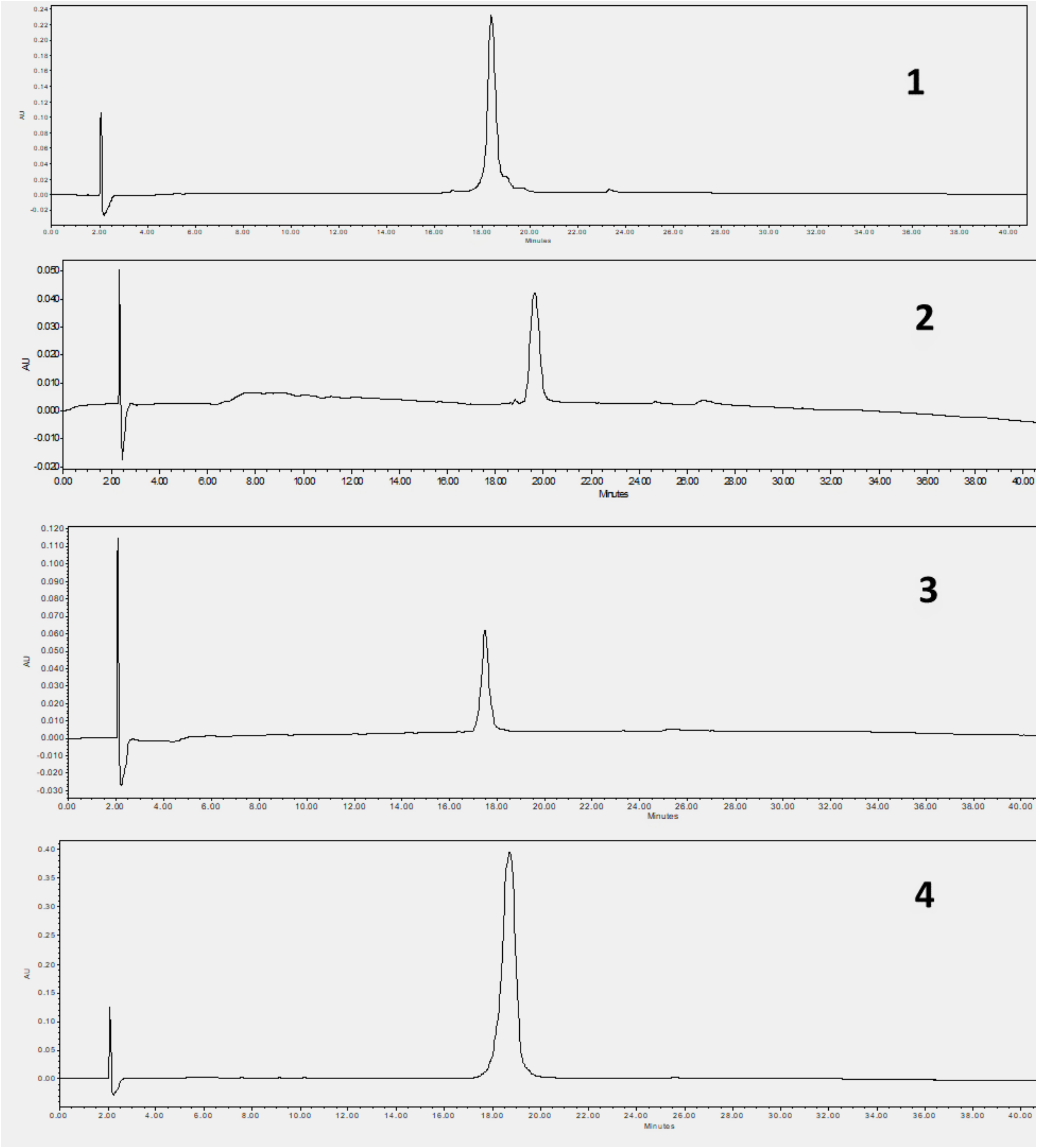

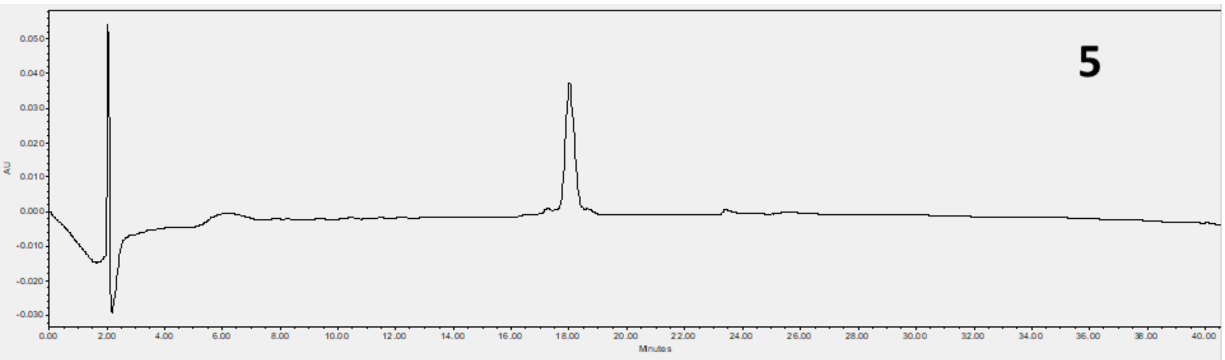
5
AU
Minutes

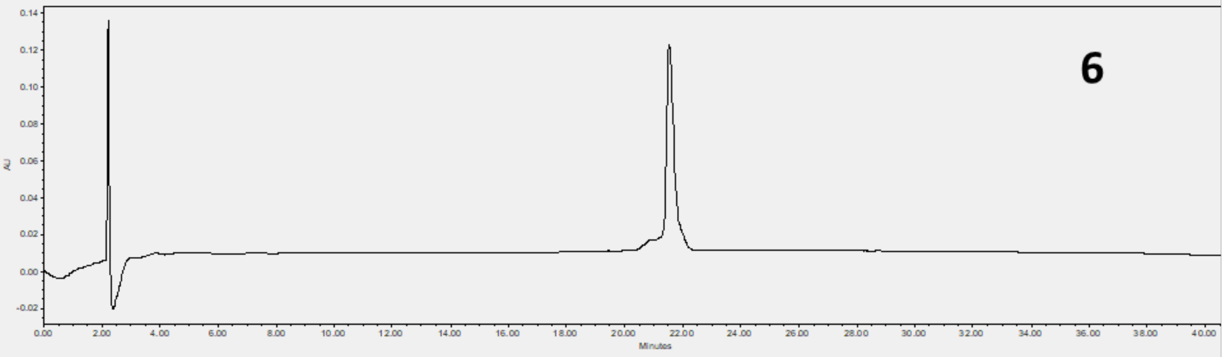
6
AU
Minutes

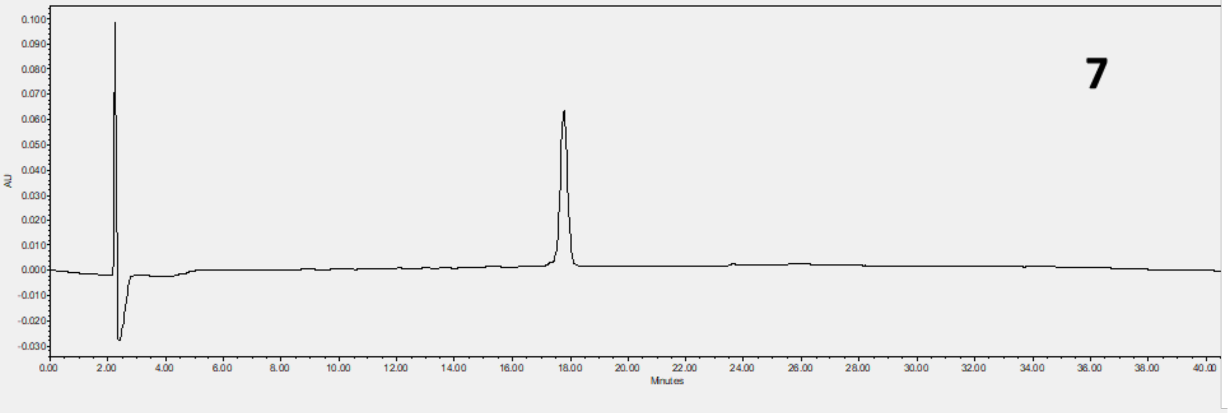
7
AU
Minutes

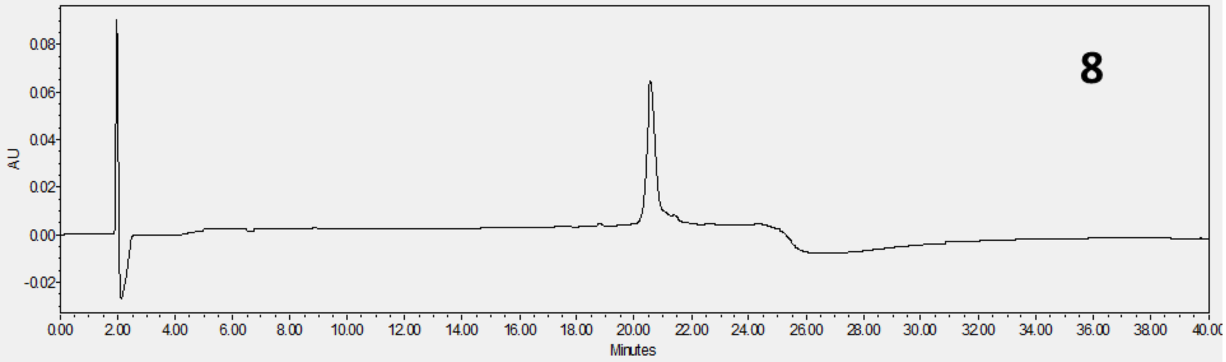
8
AU
Minutes

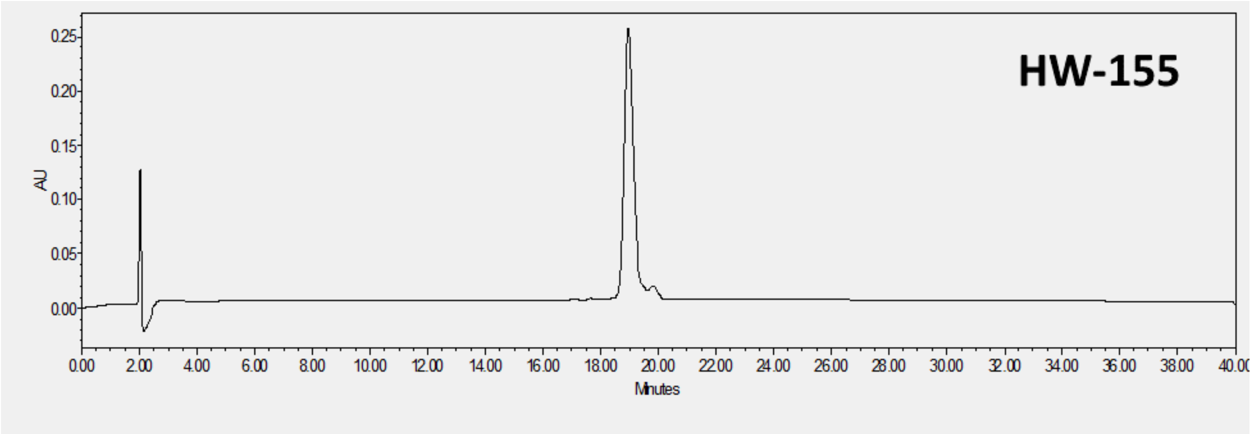

HW-155
AU
0.25
0.20
0.15
0.10
0.05
0.00
0.00 2.00 4.00 6.00 8.00 10.00 12.00 14.00 16.00 18.00 20.00 22.00 24.00 26.00 28.00 30.00 32.00 34.00 36.00 38.00
Minutes
